# Editorial: Freeze-Drying and Process Analytical Technology for Pharmaceuticals

**DOI:** 10.3389/fchem.2018.00622

**Published:** 2018-12-11

**Authors:** Davide Fissore, Timothy McCoy

**Affiliations:** ^1^Dipartimento di Scienza Applicata e Tecnologia, Politecnico di Torino, Torino, Italy; ^2^Global Biological Drug Product Development (BioDPD), Sanofi, Framingham, MA, United States

**Keywords:** thermocouple, PAT (Process Analytical Technology), freeze-drying (lyophilisation), lyophilization, monitoring

This Research Topic was focused on the freeze-drying process, a key step in the manufacturing of the pharmaceutical products due to the higher stability of solid drugs at solid state than in liquid solution, and to the fact that it allows removing the liquid from a product operating at low temperature. In 2016, 50% of the new drugs approved by the FDA were biopharmaceuticals, and about 50% of the approved biopharmaceutical drugs in the FDA and EMA lists requires freeze-drying in the manufacturing process.

Although the freeze-drying process is quite often regarded as a “soft” drying process, due to the low operating temperatures, the operating conditions have to be carefully selected aiming (i) to preserve the critical quality attributes of the product, and (ii) to minimize the duration and, thus, the cost of the process. In this framework it is particularly important to focus on product temperature, as it has to be maintained below a threshold value, that is a characteristic of the product being processed, in order to avoid the denaturation of the drug and/or the collapse of the dried cake (in case of amorphous products) or its melting (in case of crystalline products). Besides, it is necessary to detect in-line the ending point of the drying sage. Finally, it could be useful to estimate the values of the parameters of the mathematical model used to simulate, and optimize, *in silico* the process (the heat transfer coefficient between the shelf and the product in the vial, and the mass transfer resistance of the dried product to vapor flow).

Since the issue in 2004, the “Guideline for Industry PAT” motivated the researchers and the practitioners in the pharmaceutical field to develop systems, named “Process Analytical Technology” (PAT), for monitoring (and controlling) the manufacturing process. By this way the quality of the product is no longer tested at the end of the process itself, but it is evaluated in-line and, if necessary, the operating conditions are modified in order to get the desired result.

Despite the huge effort of many research groups to propose new hardware and software tools for monitoring the process, thermocouples remain widely used, and new hardware solutions have been recently proposed.

The use of thermocouples or temperature sensors as a true PAT tool has limitations, as any tool that is invasive to the product is not necessarily representative of the process. In addition, the information obtained using temperature sensors directly in the product to determine product temperature profile, and the endpoint of primary drying, depends greatly on placement. Placement can be somewhat controlled at small scale but the placement of thermocouples in the product at large scale is very challenging, due to scale and loading systems etc. There has been a variety of temperature sensors proposed in the literature, some positioned directly into the product, and some external to the product on the outside of the vial. To reduce the challenge at large scale, wireless sensors have been proposed. Apart from temperature sensors, the use of PAT technology at large scale and during commercial manufacturing has not been applied as fast as anticipated. One of the solutions to the impact of the temperature sensor positioned directly in the product would be to attempt to control ice nucleation and reduce the degree of supercooling, thereby making representative what would not have been with a temperature sensor in place.

Jiang et al. proposed a multi-point wireless system to track product temperature at several axial positions, allowing also the detection of the ending point of the process, and the rapid identification of the sublimation rate and of the heat transfer coefficient. Demichela et al. focused on the problem of the effect of incorrect thermocouples placement, as in most cases the system is assumed to track the dynamics at the vial bottom, in central position, and, due to incorrect placement of the sensor, erroneous conclusions may be driven. Therefore, beside the instrument error, this additional source of uncertainty has to be accounted for.

Monitoring of product temperature in a freeze-drying process is required as it is mandatory to properly select the operating conditions, namely the temperature of the heating shelf and the pressure in the drying chamber, in such a way that the temperature remains below a limit value that is a characteristic of the product being processed. The collapse temperature and the glass transition temperature are the two characteristics that guide the development of the process. Differential Scanning Calorimetry (DSC) and Freeze-Drying Microscopy (FDM) are the two techniques traditionally used to this purpose. The optical fibre system unit may be used as an alternative tool to detect both glass transition and collapse temperature, as well as crystallization events (Horn and Friess). It has to be remarked that this system tracks the process in one of the vials undergoing the freeze-drying process in the real equipment and, thus, more representative data are expected with respect to DSC and FDM.

Besides, there is a strong interest in monitoring what is occurring during the freeze-drying process and, in particular, microstructure formation, e.g., the ice crystal formation and growth in the freezing stage, as this is a key point to be considered due to its effect on the successive drying stage. Up to now most of the research has been focused on the ice sublimation stage, and the *in-situ* X-ray computed tomography proposed by Nakagawa et al. may be effective to assess the effect of the freezing conditions (e.g., annealing on product structure). In conclusion, it is necessary to point out that the application of PAT to lyophilization at large scale, for in-line monitoring and control, is still far from being the standard approach in the manufacturing of pharmaceutical products. In fact, PAT is nowadays considered mainly a development and scale up tool, used at lab-scale for process design and optimization. At industrial scale, PAT is looked as a tool for process monitoring, and not for control purposes. This is due to the fact that a commercial manufacturing entity prefer a known cycle time, well designed, to allow planning of manufacturing schedules, and the design of a freeze-drying cycle using well defined models, heat transfer, mass transfer, product characteristics, robust and fit to scale and load is preferred at lab scale. It is therefore necessary to strengthen the research in this field, focusing on the application at industrial scale of these devices, and pointing out the advantages, in terms of final product characteristics, that can be achieved by this way.

## Author Contributions

All authors listed have made a substantial, direct and intellectual contribution to the work, and approved it for publication.

### Conflict of Interest Statement

TM is employed by company Sanofi, Global Biological Drug Product Development (BioDPD). The remaining author declares that the research was conducted in the absence of any commercial or financial relationships that could be construed as a potential conflict of interest.

